# A Retrospective Comparison of Onabotulinum Toxin A and Incobotulinum Toxin A in Terms of Efficacy, Tolerability, Duration of Effect, and Pain on Injection Administration Site for the Treatment of Chronic Migraine

**DOI:** 10.7759/cureus.53969

**Published:** 2024-02-10

**Authors:** Scott Lucchese, Bob Daripa, Shruthi Pulimamidi

**Affiliations:** 1 Neurology/Headache Medicine, University of Arkansas for Medical Sciences, Little Rock, USA; 2 Neurology, University of Missouri School of Medicine, Columbia, USA; 3 Internal Medicine/Neurology, Singapore General Hospital, Singapore, SGP; 4 Neurology, University Hospital, Columbia, USA; 5 Internal Medicine/Neurology, Grant Medical College and Sir J.J. Group of Hospitals, Mumbai, IND; 6 Internal Medicine, University of Missouri School of Medicine, Columbia, USA

**Keywords:** pain on injection, pain, onabotulinum toxin a, incobotulinum toxin a, chronic migraine

## Abstract

Background

Onabotulinum toxin A (OnA) is a well-tolerated and effective treatment for chronic migraine (CM). However, based on research indications that incobotulinum toxin A (InA) would be equally effective, a Veterans Health Administration medical center mandated a two-year trial of InA as a more cost-effective alternative to OnA. Although InA is used for many similar indications as OnA, it is not Food and Drug Administration-approved for treating CM, and complications occurred in several patients with CM following this treatment change. We conducted this retrospective analysis to evaluate differences in the efficacy of OnA and InA and identify the reasons for the adverse effects of InA in some of these patients.

Methods

We performed a retrospective review of 42 patients who had been effectively treated with OnA and were then switched to InA. The differences between treatment responses to OnA and InA were assessed through the evaluation of pain on injection, number of headache days, and duration of action. Patients received injections at 10- to 13-week intervals. Those who reported severe pain on injection of InA were switched back to OnA.

Results

Severe burning pain on InA injection was reported by 38% of patients (nine males and seven females, i.e., a total of 16 patients out of 42 patients). One male patient reported the same degree of pain from both InA and OnA injections. A total of 66.7% of women with obesity and 83.3% of men with obesity or diabetes experienced severe pain on injection. Neither migraine suppression nor the duration of effect was significantly different between OnA and InA.

Conclusions

OnA is better tolerated than InA in the treatment of CM. InA appears to effectively suppress migraines, but some patients complain of a severe localized burning sensation during the injections. Some of these patients, all of whom were previously treated with OnA, requested to switch back to OnA. This suggested that InA is not equivalent to OnA in terms of tolerability and effectiveness. The present study found 2.38% of patients experienced an insufficient duration of effect with InA, and none with OnA. However, these lower rates may, in part, be due to variability in injection intervals in this sample, which could be because of scheduling considerations at the Harry S. Truman Veterans Health Administration Medical Center. In cases where OnA fails because of the development of antibodies, it might be reasonable to switch to InA treatment. Reformulation of InA with a pH-buffered solution may eliminate the difference in pain on injection. InA would then be a good alternative to OnA for treating CM.

## Introduction

Migraine is a potentially disabling medical condition that may be either episodic or chronic [1]. According to the diagnostic criteria of the third edition of the International Classification of Headache Disorders (ICHD 3), the occurrence of headaches on ≥15 days out of 30 with headaches on at least eight of these days meeting the ICHD criteria for migraine indicates chronic migraine (CM). A headache frequency of <15 out of 30 days is classified as episodic migraine (EM) [1].

Migraine is the second most common headache syndrome with a worldwide prevalence of approximately 14.0% [2,3]. A systematic review published in 2010 found the prevalence of CM to be between 0.9% and 5.1% [4], and a more recent review from 2020 found a similar worldwide prevalence of 4.6% [2]. Although this suggests a relatively stable incidence rate, a 2008 study found that only 20% of those who meet the diagnostic criteria for CM are properly diagnosed [5]. The annual incidence of conversion from EM to CM (known as chronification) is approximately 2.5% [6].

Migraine is the most common cause of disability [7], with CM having more detrimental effects than EM across several parameters [8]. CM is also a significant economic burden due to lost productivity and medical expenses, which were reported in 2009 to be nearly $8,000 per year per patient in the USA [9,10]. The Migraine Disability Assessment Survey (MIDAS) is a commonly used measure of the impact of migraine on patients, with higher scores indicating greater negative effects. Patients with CM have been shown to have a significantly higher mean MIDAS score than those with EM (72.57 vs. 14.49) [11]. When scores are categorized into MIDAS severity grades, the proportion of patients with CM to those with EM with the most severe disability (MIDAS grade IV) is 3 to 1 [12].

Conversion from EM to CM is associated with an increased risk of serious health issues. Comorbid psychiatric disorders, such as anxiety, depression, and bipolar disorders, are nearly twice as common in patients with CM than in those with EM [13]. Comorbid chronic pain syndromes other than migraine, such as chronic pulmonary disease, asthma, and stroke, are all approximately twice as common with CM than EM [13]. In addition to the health consequences, CM negatively affects other important aspects of life, including family, child-rearing, career, and financial status [14]. Approximately 20% of patients with CM report significant relationship problems, and 10% delay or decide against starting a family because of their illness. Approximately 60% of patients with CM have significant career problems because of their headaches, and a similar percentage report ongoing worries about financial insecurity [14].

CM headaches are more difficult to control than those in EM. This is thought to be because the high frequency of attacks precipitates changes in the brain and nociceptive structures of the head, neck, and shoulders [15]. There are currently no reliable biomarkers of CM, but changes in the brain have been observed using other physiological measures and in imaging studies [15]. A comparison of patients with CM and EM using magnetic resonance imaging has identified differences in the anterior cingulate cortex, the orbitofrontal cortex, and the volume of the hippocampus [15].

As a result of the findings of PREEMPT 1 and 2 clinical trials, the type A botulinum toxin, onabotulinum toxin A (OnA), is the only Food and Drug Administration (FDA)-approved botulinum toxin for CM treatment [16-18]. Until the release of anti-CGRP (calcitonin gene-related peptide) monoclonal antibodies, it was also the only approved CM treatment of any kind. The American Academy of Neurology gives the use of botulinum toxins for CM a level A recommendation for headache control and a level B recommendation for improvement in quality of life [19].

In the PREEMPT protocol, five units of OnA per site were injected at 31 locations on the head and neck, with up to 40 additional units available in a “follow the pain” pattern, if needed [16-18]. These sites were the locations of sensory afferent peripheral nerves of the face, scalp, neck, and shoulders, including the supraorbital, supratrochlear, auriculotemporal, and occipital nerves [20].

Treatment of CM with OnA is generally well tolerated. Side effects are relatively rare, affecting fewer than 15% of treated patients [21]. Typically, these are mild and include lateral eyebrow elevation, mild ptosis, neck and shoulder pain and stiffness, rash, and neck weakness [21].

Botulinum toxin affects the release of neuroinflammatory mediators from unmyelinated C-nerve fibers [20]. When injected, the toxin diffuses through the tissues to the local sensory peripheral nerves and enters the fibers via endocytosis [20]. Within the neuron, a light chain component of the toxin translocates into the cytoplasm and interacts with the soluble N-ethylmaleimide-sensitive factor attachment protein receptor (SNARE) complex, which is important for vesical docking in exocytosis [20]. The light chain cleaves the synaptosome-associated protein-25 kDa subcomponent of the SNARE complex, preventing vesicular fusion with the cell membrane, which blocks the release of vesical contents, such as CGRP and pronociceptive receptors [20].

CGRP is strongly implicated in the mechanism of migraines, and there is also strong evidence that the injection of botulinum toxin causes down-regulation of pronociceptive receptors. Both of these are likely involved in mitigating the dysregulation of nociceptive signal transmission thought to be associated with CM [20].

Incobotulinum toxin A (InA) is often used as an alternative to OnA [22]. OnA and InA are considered equal in effect on conditions for which both are typically used, and there is a 1:1 conversion ratio between the two [14,15]. However, InA is not FDA-approved for use in CM. To the best of our knowledge, there have been three studies of InA use for CM, all of which found it an effective treatment [23-25].

The first was a retrospective chart review of 21 patients, 12 of whom were botulinum toxin-naïve patients. An 82% response rate was reported, which was quantified as a reduction in headache frequency or intensity of at least 50%. The duration of action for InA was less than expected, with an average of 82 days and a median duration of 71 days [25].

The second of these studies was a prospective, uncontrolled evaluation using botulinum toxin treatment-naïve patients. When responsiveness was quantified as a decrease in migraine attacks of at least 50%, there was a 73% response rate. Measured as a decrease of >50% in abortive medication use, the response rate was 46% [24].

The third study was a retrospective review of the therapeutic effects and economic consequences of switching from Botox (OnA) to Xeomin (InA) [25]. They looked at several indications for botulinum toxin therapy [25]. Of the patients reviewed, 36 were treated for CM [25]. Xeomin was found to be effective and tolerable, with only two patients switching back for perceived efficacy reasons [25]. The study found InA to be 20% cheaper than OnA based on price per unit alone. When wastage was added in, the saving to each patient per annum was approximately $2000, a 38% reduction in out-of-pocket costs [25].

In our study, InA appears to effectively suppress migraines, but some patients complain of a severe localized burning sensation during the injections. Some of these patients, all of whom were previously treated with OnA, requested to switch back to OnA. This suggested that InA is not equivalent to OnA in terms of tolerability and effectiveness. To the best of our knowledge, no previous research has conducted a direct comparison of OnA and InA for effectiveness, tolerability, and early loss of effect.

This article was previously posted to the Research Square preprint server on March 14, 2023.

## Materials and methods

We conducted a retrospective, cross-sectional chart review to investigate differences in effectiveness, pain on injection, and duration of effect between InA and OnA. Data were obtained from the electronic medical records (EMR) of the Harry S. Truman Veterans Health Administration Medical Center (HSTVHAMC) in Columbia, Missouri. This study was conducted in accordance with the tenets of the Declaration of Helsinki 1975 and its later revisions. The University of Missouri Columbia Institutional Review Board and the HSTVHAMC Research and Development Committee approved this research and reviewed our consent to participate chart. This was a quality improvement project and was approved by the University of Missouri, Columbia School of Medicine Institutional Review Board.

The Veterans Health Administration (VHA) has deemed OnA and InA to be operationally identical. The VHA operational manual stipulates that the off-label administration of any botulinum toxin product is allowable when there is sufficient evidence that such use would be appropriate and effective [26]. Financial considerations and effectiveness reported in the literature led the VHA Medical Center Pharmacy and the Therapeutics Committee of the HSTVHAMC to designate InA as the preferred primary agent for the treatment of CM in place of OnA. The Arkansas State Worker's health insurance system currently has a similar policy.

Our sample consisted of US military veterans treated for CM at the HSTVHAMC between January 2016 and December 2018. The average patient age was 40 years, with a range of 26-62 years (Table 1). Two of the patients were Hispanic and one was African-American. The remaining patients were all Caucasians (Table 1). Nine patients had metabolic comorbidities, including type 2 diabetes mellitus and obesity (Table 2).

**Table 1:** Demographic data The male-to-female ratio was 2.23. The total number of subjects was 42.

Age (years)	
Range	25-62
Average	40
Sex	
Male	69.0% (n = 29)
Female	31.0% (n = 13)
Ethnicity	
White	92.8% (n = 39)
Hispanic	4.8% (n = 2)
African-American	2.4% (n = 1)

**Table 2:** Metabolic comorbidities Obesity is considered when body mass index (BMI) ≥ 30.

Medical condition	Male	Female
Diabetes mellitus	2	0
Obesity	2	5
Obesity and diabetes mellitus	2	0

The sample consisted of 42 patients, 13 women and 29 men, who met the following inclusion criteria: those who were treated with OnA according to the standard PREEMPT protocol with a good response before switching to InA (good response being a reduction of ≥50% in the frequency of migraine attacks below prior baseline), met the ICHD 3 diagnostic criteria for CM, were treated with at least one injection cycle of InA, and had no break in botulinum treatment through the course of the study. Exclusion criteria were a headache diagnosis other than CM, secondary headaches, previous failure to respond to OnA, and failure to complete at least five injection sets without a greater than one-month break-in sequence. There were eight injection cycles per patient.

Any problems during an injection visit were recorded in the patient's EMR. After an injection sequence, patients were routinely asked to rate their pain from 1 to 10 on the Visual Analog Scale [27]. A rating of ≥7 was recorded as an adverse drug reaction to the toxin. For reporting purposes, pain ratings were categorized by severity, with ratings of 1-2 indicating mild pain, 3-6 indicating moderate pain, and ≥7 indicating severe pain. Similarly, the effectiveness of each injection set, based on headache diaries and patient reports, was recorded in each patient's EMR. During reviews, changes in headache frequency of more than two additional headache days per week were recorded. An increase in headache frequency over the last two to three weeks of the injection cycle only was also reported in the EMR. The three parameters reviewed were localized pain on injection, effectiveness, and sufficient longevity of effect between cycles. These were recorded in anonymized files before analysis.

Effectiveness after switching to InA was considered good if there was an increase in headache frequency of <10%. Pain on injection was measured on the Visual Analog Scale [27]. The duration of effect was determined by an increase in attack frequency by two headaches per week over baseline beginning ≥ two weeks before the next scheduled injection set.

## Results

Pain on injection

The extent of injection site pain differed between the two agents. When treated with InA, 17 patients (40.5%) reported severe pain (Table 3).

**Table 3:** Comparison of the effectiveness, tolerability, and duration of the effect of incobotulinum toxin A and onabotulinum toxin A Classification of pain as moderate or severe was based on a numeric rating scale of pain intensity. Only severe intensity of pain on injection was found to be statistically significant (p < 0.05). No other measure reached significance. The total number of subjects was 42.

S. No.	Results		Incobotulinum toxin A		Onabotulinum toxin A	
1	Effectiveness (reduction in migraine attack frequency)		n	%	n	%
	(a)	Good (at least 50% reduction)	39	92.9	41	97.6
	(b)	Poor	3	7.1	1	2.4
2	Tolerability (pain at the injection site)					
	(a)	Severe intensity	17	40.5	1	2.38
	(b)	Moderate intensity	2	4.8	2	4.8
	(c)	No pain	23	54.8	39	92.9
3	Duration of effect					
	(a)	12 weeks	39	92.9	40	95.2
	(b)	Early wear-off (≥1 week before the next scheduled injection date)	3	7.1	2	4.8

One patient (2.38%, male) reported severe pain from both OnA and InA injections. One patient (2.38%) reported severe pain with InA but moderate pain with OnA, and one patient (2.38%) reported moderate pain with both OnA and InA. Seven women (53%) and nine men (31%) reported severe pain with InA only (Table 4). Moderate injection pain was reported with InA only by one patient (2.38%). In all cases, the pain was reported as burning in character.

**Table 4:** Comparison of pain on injection administration by gender The total number of subjects was 42. OnA: onabotulinum toxin A; InA: incobotulinum toxin A; n: number of subjects; a/w: associated with.

Gender	Intensity of pain		Subjects experiencing pain a/w InA injection only	Subjects experiencing pain a/w OnA injection only		Subjects experiencing pain a/w both InA and OnA injections
Both male and female						
	Severe	16 (38.09%)			0	1 (2.38%)
	Moderate	0			1 (2.38%)	1 (2.38%)
Only males (the total number of male subjects was 29)						
	Severe		9 (31.03% of total males or 21.42% of total subjects)	0		1 (3.44% of total males)
	Moderate		1 (3.44% of total males)	0		0
Only female (the total number of female subjects was 13)						
	Severe		7 (53.84% of total females or 16.66% of total subjects)	0		0
	Moderate		0	0		1 (7.69% of total females)

When considered by comorbidity, two of three women with obesity (66.7%) reported severe pain on injection and five of six (83.3%) men with metabolic comorbidities (obesity or diabetes) reported severe pain on injection with InA only (Table 5). One man who had psoriasis reported excess pain with InA only. Patients without metabolic comorbidity had 10 of 31 (32.2%) patients experience severe pain on injection (Table 5).

**Table 5:** Comparison of pain on injection administration between patients with and without metabolic comorbidity No woman had DM in this review. One man had psoriasis, which is not mentioned in the above table. DM: type 2 diabetes mellitus; OnA: onabotulinum toxin A; InA: incobotulinum toxin A; a/w: associated with.

Metabolic comorbidity	Pain a/w InA only	Pain a/w OnA only	Pain a/w InA and OnA
Males without comorbidity (n = 23)	6	0	0
Females without comorbidity (n = 10)	4	0	0
DM in men	2	0	0
Obesity in men	2	0	0
Obesity in women	3	0	0
DM and obesity in men	1	0	0

Effectiveness

A poor response to both InA and OnA was seen in only one patient (2.38%, male), who had previously responded well to OnA; this was also the patient who reported severe injection pain with both InA and OnA. Poor responses to InA alone were seen in two patients (4.76%), one man and one woman. None of the patients responded poorly to OnA only.

Longevity of effect

Patient reports of the longevity of effects were nearly identical for both products, with two patients (4.76%) reporting insufficient effect duration for both OnA and InA (one man and one woman) and one patient (2.38%) for InA only (male). Both the patients who experienced insufficient effect duration for both OnA and InA also reported severe injection pain with InA but not OnA.

## Discussion

Our findings indicated that InA, and to a lesser extent OnA, can cause severe pain at the injection site. Although these results are difficult to generalize because of the retrospective nature of the study and the small sample size, they are consistent with prior reports of pain with botulinum toxin injections [28]. They, therefore, highlight the need for larger, more definitive studies. The results of such work may influence whether facilities and funding bodies recommend InA use for the treatment of CM and other indicated health issues, such as facial spasms, jaw dystopia, and depression [25], not considered in our study.

OnA and InA each have their own benefits. In OnA, the toxin molecules are bound to nontoxic complexing proteins, whereas in InA, they are not [29]. There also appears to be a lower risk of patients developing InA antibodies [29]. InA does not require refrigeration and is available in smaller dosage vials, making it more cost-effective [25].

Our results suggest that OnA is better tolerated than InA in the treatment of CM. Nearly half (16 patients, 38.09%) of the patients reviewed experienced severe injection pain in response to InA, whereas none was reported with OnA only. The patient who suffered severe pain with both toxins was also unresponsive to both treatments, although he had benefited from OnA treatment before his first InA injection set. It is possible that there was a confounding issue at play in his case.

The burning pain associated with InA injections may be related to its lack of complexing proteins. However, in other formulations, these proteins may provide some antinociceptive benefits, such as a buffering system, to prevent rapid changes in pH or alterations in the osmotic pressure of the tissue surrounding the injection site. Both botulinum toxins have acidic pH in their standard formulations. The reported pH of OnA is 6.09 and that of InA is 5.81 [28]. Reformulation with a buffering solution has been reported to reduce injection site pain with both of these toxins, with pain reduction found in 47% of OnA patients and 76% of InA patients and no reductions in effectiveness [28]. Given the financial advantages of InA, further research into such reformulations is warranted because the elimination of excessive injection pain would make it the preferred agent.

One previous study has found both of these medications to lose efficacy fewer than 10 or 11 weeks after injection in up to 25% of cases [30]. Although the present study also found evidence of this, only 4.76% of patients experienced an insufficient duration of effect with both toxins, 2.38% with just InA, and none with just OnA. These lower rates may, in part, be due to variability in injection intervals in this sample. Because of scheduling considerations at HSTVHAMC, patients were often injected one to two weeks earlier than the typical 12-week interval.

## Conclusions

OnA is better tolerated than InA in the treatment of CM. InA appears to effectively suppress migraines, but some patients complain of a severe localized burning sensation during the injections. Some of these patients, all of whom were previously treated with OnA, requested to switch back to OnA. This suggested that InA is not equivalent to OnA in terms of tolerability and effectiveness.

The above medications tend to lose efficacy fewer than 10 or 11 weeks after injection. The present study found 2.38% of patients experienced an insufficient duration of effect with InA, and none with OnA. Although these lower rates may, in part, be due to variability in injection intervals in this sample, which could be because of scheduling considerations at HSTVHAMC, patients were often injected one to two weeks earlier than the typical 12-week interval. In cases where OnA fails because of the development of antibodies, it might be reasonable to switch to InA treatment. Reformulation of InA with a pH-buffered solution may eliminate the difference in pain on injection. InA would then be a good alternative to OnA for treating CM.
